# Lack of 17β-estradiol reduces sensitivity to insulin in the liver and muscle of male mice

**DOI:** 10.1016/j.heliyon.2018.e00772

**Published:** 2018-09-11

**Authors:** Katsumi Toda, Akiko Toda, Masafumi Ono, Toshiji Saibara

**Affiliations:** a Department of Biochemistry, Kochi University School of Medicine, Nankoku, Kochi 783-8505, Japan; b Department of Gastroenterology and Hepatology, Kochi University School of Medicine, Nankoku, Kochi 783-8505, Japan

**Keywords:** Health sciences, Biological sciences, Endocrinology, Metabolism, Physiology, Biochemistry

## Abstract

The importance of estrogens for glucose homeostasis has been demonstrated by clinical, pharmacological, and experimental studies. Male mice lacking the aromatase gene (ArKO mice), which encodes an enzyme involved in estrogen synthesis, develop glucose- and insulin-intolerance. However, it remains unclear whether insulin signaling is actually impaired in the liver and muscle of ArKO mice. We examined the effects of estrogen-deficiency on insulin signaling by quantifying phosphorylation levels of protein kinase B (Akt) in the liver and muscle and by examining the expression levels of insulin-target genes in the liver. Insulin administration enhanced phosphorylation levels of Akt in the liver and muscle of wild-type (WT) mice, ArKO mice, and ArKO mice supplemented with 17β-estradiol (E2), but insulin was less effective in ArKO mice. Gene expression analysis revealed that alterations induced by insulin in WT liver were also observed in ArKO liver, but the degree of altered expression in a subset of genes was smaller in ArKO mice than in WT mice. E2 supplementation improved the insulin responses of some genes in ArKO mice. Thus, these findings suggest that insulin signaling in the liver and muscle of ArKO mice is less efficient than in WT mice, which contributes to whole-body glucose intolerance in ArKO mice.

## 1 Introduction

Metabolic abnormalities, such as dyslipidemia and hyperglycemia, are a major health concern worldwide. Management of plasma glucose levels, which fluctuate within a narrow range, is one way to prevent the development of metabolic disorders. Maintenance of glucose homeostasis is achieved by functional interactions among various organs including the pancreas, liver, skeletal muscle, adipose tissue, and brain. Studies using genetically engineered murine models found that the liver has a vital role in overall glucose disposal and that hepatic insulin resistance significantly impairs whole-body glucose homeostasis (Michael et al., 2000; Saltiel and Kahn, 2001; Biddinger and Kahn, 2006).

Estrogens have beneficial effects on metabolic homeostasis in addition to their roles in reproductive functions (Nuutila et al., 1995; Carr, 2003; Herrmann et al., 2002; Maffei et al., 2004; Riant et al., 2009). The effects of estrogens on the regulation of insulin action and glucose metabolism have been underscored by studies of genetically engineered murine models, such as estrogen receptor (ER) α gene (*ESR1*) knockout mice (ERαKO mice) (Heine et al., 2000) or mice deficient in the aromatase gene (*Cyp19a1*), which encodes an enzyme responsible for estrogen synthesis (ArKO mice) (Nemoto et al., 2000; Takeda et al., 2003; Van Sinderen et al., 2014). ERαKO mice develop glucose intolerance and have decreased insulin sensitivity because of hepatic insulin resistance (Bryzgalova et al., 2006; Zhu et al., 2014). ArKO male mice are obese and develop glucose intolerance and insulin resistance, but supplementation with 17β-estradiol (E2) improves systemic insulin sensitivity (Takeda et al., 2003). Furthermore, E2 has anti-diabetic and weight-lowering effects in ovariectomized murine models and spontaneous rodent models of type 2 diabetes (Bailey and Ahmed-Sorour, 1980; Louet et al., 2004). Although the beneficial effects of E2 on glucose and lipid metabolism are well established, how E2 affects the hepatic responses to insulin in ArKO mice has not been studied. A clearer understanding of the molecular mechanisms that disrupt hepatic responses to insulin in estrogen-deficient states is essential for optimal management of estrogen-depleted states in humans to prevent long-term morbidities. Thus, we examined the responses of insulin-target tissues, such as liver and muscle, using matured ArKO male mice, which develop systemic insulin resistance (Takeda et al., 2003; Van Sinderen et al., 2014).

Insulin signaling is initiated by the intrinsic tyrosine kinase activity of the insulin receptor, which catalyzes tyrosine phosphorylation of IRS-1, IRS-2, and other endogenous substrates. This in turn triggers signaling pathways that activate downstream serine-specific protein kinases that mediate insulin action, including protein kinase B/Akt, to control cellular metabolism (Lu et al., 2012; Taniguchi et al., 2006). Akt activation is elicited by phosphorylation in its kinase domain at Thr^308^ by phosphoinositide-dependent protein kinase 1 (PDPK1) following insulin stimulation. Furthermore, full Akt activation is associated with a second phosphorylation at Ser^473^ in the hydrophobic motif domain by mTORC2 (Sarbassov et al., 2005). Activated Akt then phosphorylates a myriad of protein substrates with diverse subcellular localizations (Manning and Cantley, 2007), though it seems to be an important future area of research whether Akt phosphorylated at both or one of the sites modifies different downstream substrates (Manning and Toker, 2017). We examined phosphorylation levels of Thr^308^ and Ser^473^ in Akt 10 or 60 min after insulin administration as proximal insulin signaling events to determine whether ArKO mice respond to insulin properly in two insulin target tissues, liver and muscle. In addition to phosphorylation levels, we examined transcriptional hepatic responses to insulin using newly identified insulin-target genes as indicators because not all insulin-regulated processes become equally resistant to insulin in insulin-resistant states. We further investigated whether E2 restored altered insulin-responses of ArKO mice similar to those of WT mice. We found that liver and muscle were less sensitive to insulin in ArKO mice than in WT mice.

## 2 Materials and methods

### 2.1 Experimental animals

The experiments using animals were conducted according to established animal welfare guidelines and experimental protocols approved by Animal care committee of Kochi University (Approved No. K-00016). Mice at 5–6 months of age were used, maintained on a 12-h light/dark cycle at 22–25 °C, and given water and rodent chow (CE-2, Oriental Yeast Ltd., Tokyo, Japan). ArKO mice in a C57BL/6J genetic background were generated in our laboratory previously (Toda et al., 2001, Toda et al., 2008), and heterozygous offspring were mated to obtain ArKO mice. To examine the effects of E2 on insulin responses, a group of ArKO mice at 5 months of age were fed for 30 d a chow diet containing 10 ppm E2 (ArKO/E2 mice), which was prepared by dissolving 10 mg E2 (98%, purity minimum, Sigma-Aldrich Corp., Tokyo, Japan) in 100 mL acetone and adding it to 1 kg rodent chow (Toda et al., 2004). Fed mice injected with phosphate buffered saline (PBS) were assigned as fed mice. After mice fasted for 19 h, an intraperitoneal (ip) injection of PBS (fasted mice) or insulin (recombinant regular human insulin, Novolin R, Novo Nordisk Inc., NJ, USA) at a dose of 0.75 mU/g of body weight (insulin-treated mice) was administered. Liver (∼0.1 g) and gastrocnemius (∼0.3 g) were dissected 10 or 60 min after injection, snap-frozen in liquid nitrogen, and stored at −80 °C until use.

### 2.2 Measurement of glucose, triacylglycerol, cholesterol, insulin, and glucagon

Tail blood was collected via vein nicks of mice for measurement of blood glucose levels. Serum was prepared from blood collected by cardiac puncture, and stored at −20 °C. The concentrations of serum triacylglycerol and serum cholesterol were measured by colorimetric methods using the Triglyceride E-Test and Cholesterol T-Test, respectively (Wako Pure Chemical Industries Ltd., Osaka Japan). Serum insulin and serum glucagon levels were determined by using enzyme-linked immunoassay kits (BioVendor Laboratorni medicina a.s., Modrice Czech Republic, and Mercodia AB, Uppsala, Sweden, respectively). Blood glucose concentration was measured with Glutest Ace and Glutest Sensor (Sanwa Kagaku Kenkyusho Co., Nagoya, Japan).

### 2.3 Western blot analyses

To prepare tissue lysates, a portion of liver (∼0.1 g) from each mouse was sonicated in 0.1 mL of homogenization buffer consisting of 50 mM Tris-HCl (pH 7.4), 0.1 mM EDTA, 0.1 mM EGTA, 0.1% (w/v) SDS, a protease inhibitor cocktail (Roche Diagnostics GmbH, Germany), and a phosphatase inhibitor cocktail (Pierce, Rockford, IL, USA). Frozen gastrocnemius (∼0.3 g) was homogenized in 0.4 mL of homogenizing buffer with a TaKaRa BioMasher Standard (TAKARA BIO INC. Shiga 520-2193, Japan) and sonicated. Whole tissue lysates were transferred to new tubes and used to analyze protein phosphorylation levels. Protein concentrations of the lysates were quantified using a bicinchoninic acid protein assay kit (Thermo Scientific, Rockford, IL, USA).

Lysates (25 μg of protein/lane) were separated by 10% (w/v) SDS-PAGE and transferred to polyvinylidene difluoride membrane filters (Bio-Rad Laboratories, Inc., CA, USA). Membrane filters were blocked with 5% (w/v) bovine serum albumin solution (Wako Pure Chemical Industries, Osaka, Japan) in 20 mM Tris-HCl (pH 7.6), 0.14 M NaCl, and 0.05% (w/w) TWEEN^®^20 (Sigma-Aldrich, St. Louis, MO, USA) (TBS-T) at 4 °C for more than 2 h. Membranes were incubated with primary antibodies (Akt, pAkt^Thr308^, or pAkt^Ser473^) in Immunoshot® reagent 1 (Cosmo Bio Co., Ltd., Tokyo, Japan) overnight at 4 °C with continuous shaking. Membranes were washed with TBS-T, incubated in goat anti-rabbit horseradish peroxidase-conjugated IgG secondary antibody for 3 h at 25 °C, washed with TBS-T, and incubated for 5 min with Luminata™ Crescendo Western HRP Substrate (Millipore Corporation, MA, USA). Immuno-reacted bands were visualized by a luminescent image analyzer, ImageQuant™ LAS 4000 mini (GE Healthcare Bio-Sciences AB, Uppsala, Sweden), and quantified by Image Gauge Ver. 3.2 (FUJIFILM Corp. Tokyo), in which the intensities of immuno-reacted bands were expressed in arbitrary units. Primary antibody suppliers and antibody dilution ratios are listed in Table 1.

**Table 1:** Antibodies used in the study.

	Abbreviation	Dilution	From	Cat. No.	RRID
phospho-Akt (Thr308)	pAkt^T308^	1:1000	Cell Signaling Technology, Beverly, MA, USA	4056	AB_331163
phospho-Akt (Ser473)	pAkt^S473^	1:1000	Cell Signaling Technology, Beverly, MA, USA	4060	AB_2315049
Akt	Akt	1:1000	Cell Signaling Technology, Beverly, MA, USA	9272	AB_329827
Peroxidase-labeled antibody to rabbit IgG (H + L)		1:20000	SeraCare Life Sciences, Milford, MA, USA	074-1516	

### 2.4 RNA preparation and real-time quantitative PCR (RT-qPCR) analysis

Total hepatic RNAs were prepared individually using NucleoSpin® RNAII (Macherey-Nagel GmbH & Co., Düren, Germany). RNA (0.5 μg) was reverse-transcribed using a PrimeScript^®^RT reagent kit with gDNA Eraser (Takara Bio Inc., Shiga, Japan). Quantitative PCR analysis was performed with total reverse-transcribed RNA (16 ng equivalent) and 10 μM of each primer in a final volume of 20 μL using SYBR^®^ *Premix Ex* Taq™ II in a light cycler instrument (Takara Bio Inc., Shiga, Japan). After initial denaturation (95 °C for 30 s), 40 cycles of amplification (95 °C for 5 s and 60 °C for 20 s) were performed. Gene expression levels were calculated by the comparative CT method. Ribosomal protein large subunit 19 (Rpl19) mRNA levels from each sample were used as an internal control to normalize mRNA levels. We divided expression levels of each gene in insulin-treated mice with the mean expression level of mice treated with saline alone to obtain fold-differences. Gene-specific primer sequences are listed in Table 2.

**Table 2:** Primers used in qRT-PCR analysis.

	Gene symbol	Gene accession	Forward	Reverse	Size	PrimerBank ID
Angiopoietin-like 4	Angptl4	NM_020581	CATCCTGGGACGAGATGAACT	TGACAAGCGTTACCACAGGC	136	10181164a1
Arrestin domain containing 3	Arrdc3	NM_001042591	CAGAGGTTGTAACGGAGGAAC	GGGGCAGGAACCGAAACTC	123	28972730a1
B cell translocation gene 2, anti-proliferative	Btg2	NM_007570	GGACGCACTGACCGATCATTA	GATACAGCGATAGCCAGAACC	76	84875511c2
C-type lectin domain family 2, member d	Clec2d	NM_053109	GGTTTGACAACCAGGATGAGC	TCTCCCCGGATGGGAATCG	148	16716407a1
Dual specificity phosphatase 1	Dusp1	NM_013642	TTTGAGTTTGTGAAGCAGAGG	GTGGGGTGGACGGGGATGGAA	197	-
Fatty acid synthase	Fasn	NM_007988	GCTCATGGGTGTGGAAGTT	AGCTGGGTTAGGGTAGGAC	231	-
Fibroblast growth factor 21	Fgf21	NM_020013	GTGTCAAAGCCTCTAGGTTTCTT	GGTACACATTGTAACCGTCCTC	123	146134956c1
Fibrinogen-like protein 1	Fgl1	NM_145594	CTTCGTCCTGGTCGCCATT	TCCCGCAAGCAGTTCTCAC	75	262331535c1
Glucose-6-phosphatase, catalytic	G6pc	NM_008061	TGAAACTTTCAGCCACATCCG	GCAGGTAGAATCCAAGCGCGAA	101	-
Inhibin beta-E	Inhbe	NM_008382	AAGATCCGAGCTAATGAACCTGG	GGTCTCGCCTACAACATAAGGG	91	162139830c3
Interleukin 1 beta	Il1b	NM_008361	CTGTGACTCATGGGATGATGATG	CGGAGCCTGTAGTGCAGTTG	75	118130747c3
Insulin receptor substrate 2	Irs2	NM_001081212	CTGCGTCCTCTCCCAAAGTG	GGGGTCATGGGCATGTAGC	124	3661525a1
Malic enzyme 1, NADP(+)-dependent, cytosolic	Me1	NM_008615	GTCGTGCATCTCTCACAGAAG	TGAGGGCAGTTGGTTTTATCTTT	102	199019a1
Nuclear receptor subfamily 4, group A, member 1	Nr4a1	NM_010444	CTGTCCGCTCTGGTCCTC	AATGCGATTCTGCAGCTCTT	84	-
Orosomucoid 2	Orm2	NM_011016	CAACATCACCATAGGCGACCC	ATTTCCTGCCGGTAATCAGGG	108	112181191c1
Pyruvate kinase liver and red blood cell	Pklr	NM_013631	TACCACCGCCAGTTGTTTG	GCGGCCAGTCTTTGTCAGC	144	-
Phosphoenolpyruvate carboxykinase 1, cytosolic	Pck1	NM_011044	TGACAGACTCGCCCTATGTG	CCCAGTTGTTGACCAAAGGC	153	118130217c3
RasGEF domain family, member 1B	Rasgef1b	NM_145839	ACCGAAACCTCTACCAGTCC	CCAAGTGTTGGATAAGGGCTTC	102	22003890a1
Regulator of calcineurin 1	Rcan1	NM_001081549	TTGTGTGGCAAACGATGATGT	CCCAGGAACTCGGTCTTGT	189	31542574a1
Regulator of G-protein signaling 16	Rgs16	NM_011267	CGAGTGGGCCAGTAAGCATAA	GCAAATCGAAAGACTCTCTCCA	77	190684664c2
Stearoyl-Coenzyme A desaturase 1	Scd1	NM_009127	CCGGAGACCCCTTAGATCGA	TAGCCTGTAAAAGATTTCTGCAAACC	89	-
Serine (or cysteine) peptidase inhibitor, clade E, member 1	Serpine1	NM_008871	GTGAATGCCCTCTACTTCAGTG	GCTGCCATCAGACTTGTGGAA	90	170172561c3
Salt inducible kinase 1	Sik1	NM_010831	CAGGTGCTAGGGATCATGCAG	GGAGGTAGTAAATGGCGGCAA	103	110815829c2
Transformation related protein 53 inducible nuclear protein 1 transcript variant 2,	Trp53inp1	NM_001199105	GTTGACTTCATAGATACCTGCCC	GTGTGCTCTGCTGAGGACTC	89	312283686c1
Thioredoxin interacting protein	Txnip	NM_001009935	TCAATACCCCTGACCTAATGGC	TTCTGTCAATTCGAGCAGAGAC	108	118131130c3
Ribosomal protein L19	Rpl19	NM_001159483	TACCGGGAATCCAAGAAGATTGA	AGGATGCGCTTGTTTTTGAAC	89	226958656c3

### 2.5 Microarray analysis

A pool of total hepatic RNA prepared individually from fasted WT mice injected with PBS (n = 7) or insulin (n = 7) was used for microarray analysis. After examining RNA integrity using the 2100 Agilent Bioanalyzer system (Agilent Technologies, CA, USA), a portion of the mixture (100 ng) was used to generate amplified and biotinylated sense-strand DNA using a GeneChip® WT PLUS Reagent Kit for analysis with Mouse Gene 2.0 ST Array (Affymetrix Japan K.K., Tokyo, Japan). Average signal intensities for each probe set within the arrays were calculated by and exported from Affymetrix® Expression Console™ software (ver. 1.3.0) using the robust multichip average (RMA) method. Microarray data have been deposited in the Gene Expression Omnibus (www.ncbi.nlm.nih.gov/geo, accession number GSE111407).

### 2.6 Statistical analysis

Data were analyzed with a PSI-Plot software (Poly Software International Inc., NY, USA). All data are presented as mean ± SEM. Differences were considered significant when the p value was less than 0.05.

## 3 Results

### 3.1 Serum parameter

**Fig. 1:**
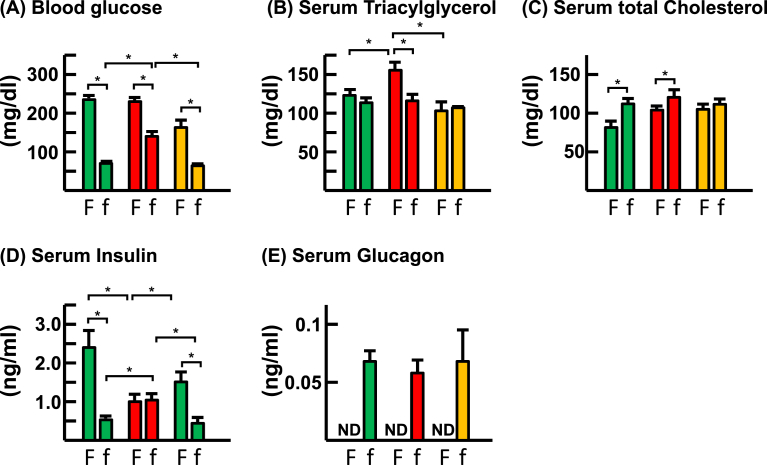
Measurement of metabolic parameters. Various metabolic parameters were measured in blood or serum samples collected from fed (F) or fasted (f) WT (green bar), ArKO (red bar), and E2-supplemented ArKO (orange bar) mice. (A) Blood glucose (n = 12) (B) serum triacylglycerol (n = 6), (C) serum cholesterol (n = 6), (D) serum insulin (n = 8), and (E) serum glucagon of fasted mice (n = 5) concentrations were measured. *, p < 0.05.

The blood glucose level after 19 h fasting is significantly high in ArKO mice as compared to those of WT and ArKO/E2 mice (Fig. 1A). Serum triacylglycerol contents in fed state were significantly elevated in ArKO mice than WT and ArKO/E2 mice, while the contents in fasted mice were not different among experimental groups (Fig. 1B). Serum total cholesterol contents in fed mice were not different among experimental groups, whereas those were elevated after fasting in WT and ArKO mice, but not in ArKO/E2 mice (Fig. 1C). Serum insulin level is significantly lower in fed ArKO mice, but it is higher in fasted ArKO mice as compared to those of WT and ArKO/E2 mice (Fig. 1D). Serum glucagon levels in fasted mice were not different among experimental groups (Fig. 1E). The high levels of blood glucose and serum insulin in the fasted ArKO mice indicate its development of insulin resistant phenotype. In addition, this phenotype of ArKO male mice is corrected with E2 supplementation carried out under current experimental conditions.

### 3.2 Western blot analysis of phosphorylation levels in the liver

**Fig. 2:**
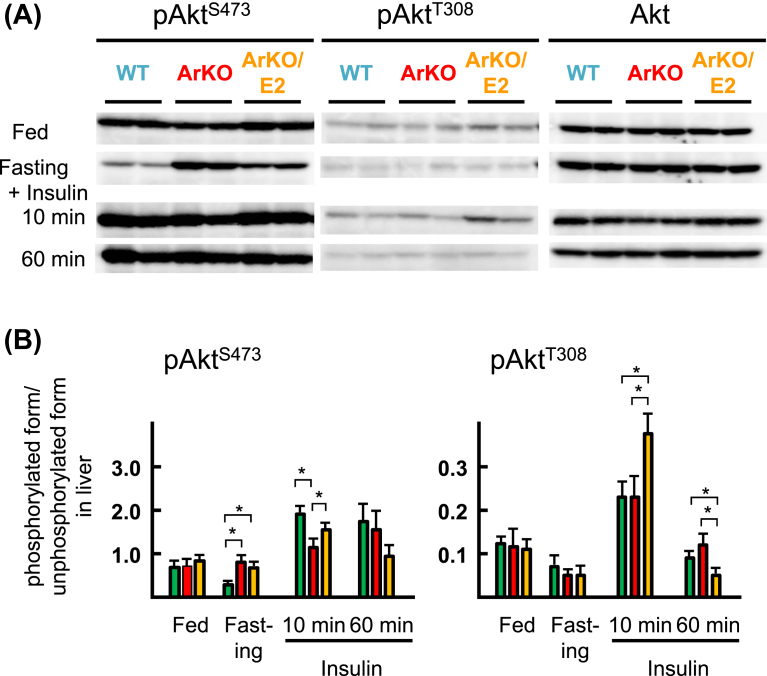
Western blot analyses of basal and insulin-stimulated phosphorylation of Akt in the liver. Phosphorylation levels of Akt (Ser^473^ and Thr^308^) and total Akt were analyzed from protein extracts from the liver of WT (green bar), ArKO (red bar), and ArKO/E2 mice (orange bar) fed, fasting for 19 h, and at 10 or 60 min after intraperitoneal injection of insulin into fasted mice. (A) Representative results of western blot analyses are shown. (B) Phosphorylated levels of Akt were normalized to total Akt (n = 6 for each group). *p < 0.05; compared among WT, ArKO, and ArKO/E2 mice. Unaltered blots are shown in Supplementary Fig. 1.

Differences in the phosphorylation level of Ser^473^ in Akt were undetectable among WT, ArKO, and ArKO/E2 mice in the fed state (Fig. 2). After fasting, the phosphorylation level markedly decreased in WT mice, but not in ArKO mice. The level decreased in ArKO/E2 mice after fasting, but the degree was smaller than that in the WT mice. When fasted mice received insulin, phosphorylation levels increased in all three experimental groups. However, the degree of the increase was significantly lower in ArKO mice than in WT and ArKO/E2 mice. The phosphorylation level was retained 60 min after insulin administration without significant differences among the experimental groups.

Phosphorylation levels of Thr^308^ in Akt were less apparent than those of Ser^473^ in the liver of fed mice, and levels further decreased after fasting. Insulin administration increased the level markedly in all experimental groups, and the increase was especially apparent in the liver of ArKO/E2 mice. In contrast to phosphorylation of Ser^473^, the level of Thr^308^ decreased precipitously 60 min after insulin administration. Although the bands detected were faint, the phosphorylation levels in ArKO/E2 mice were significantly lower than those in WT or ArKO mice.

In summary, Western blot analyses of livers revealed that insulin increases the Akt phosphorylation levels of Ser^473^ and Thr^308^ in WT, ArKO, and ArKO/E2 mice. There were significant differences in the phosphorylation levels of Ser^473^ between WT and ArKO mice: the phosphorylation level in the fasted state in ArKO mice is higher than in WT mice and the level after insulin supplementation is lower in ArKO mice than in WT mice.

### 3.3 Western blot analysis of phosphorylation levels in the muscle

**Fig. 3:**
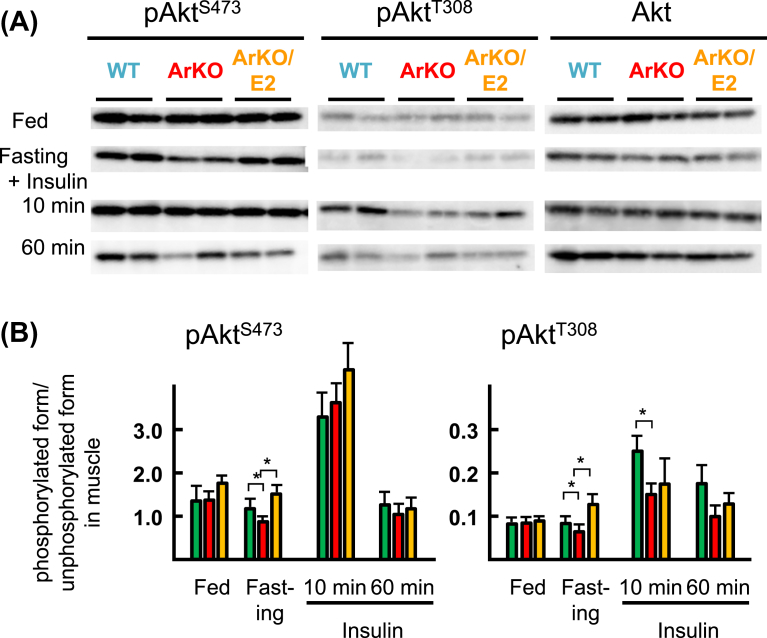
Western blot analyses of basal and insulin-stimulated phosphorylation of Akt in the muscle. Phosphorylation levels of Akt (Ser^473^ and Thr^308^) and total Akt were analyzed from protein extracts from the gastrocnemius of WT (green bar), ArKO (red bar), and ArKO/E2 (orange bar) mice fed, fasting for 19 h, and at 10 or 60 min after intraperitoneal injection of insulin into fasted mice. (A) Representative results of western blot analysis are shown. (B) Phosphorylated levels of Akt were normalized to total Akt (n = 6 for each group). *p < 0.05; compared among WT, ArKO, and ArKO/E2 mice. Unaltered blots are shown in Supplementary Fig. 2.

As observed in the liver (Fig. 2), high levels of phosphorylation of Ser^473^ in Akt were detected in the muscle in the fed state regardless of genotypes and E2 supplementation (Fig. 3). However, the phosphorylation level decreased after fasting more markedly in ArKO mice than in WT and ArKO/E2 mice, which was different from the levels in the liver. Insulin administration increased the phosphorylation level regardless of genotype or E2 treatment 10 min after injection. The phosphorylation level of Ser^473^ in Akt decreased 60 min after injection, which was also different from the liver, where levels were maintained.

Low levels of phosphorylation of Thr^308^ in Akt were detected compared to those of Ser^473^ in the muscle in the fed state. The phosphorylation levels after fasting were lower in ArKO muscle than in WT and ArKO/E2 mice. Insulin supplementation increased the level markedly, but less in ArKO mice than in WT mice. Similar to the phosphorylation level of Ser^473^, the level of Thr^308^ decreased 60 min after insulin supplementation.

Western blot analysis in muscle revealed that insulin increased the phosphorylation levels of Thr^308^ in WT mice more than in ArKO mice, which suggests that the muscle of ArKO mice was less sensitive to insulin than those of WT and ArKO/E2 mice.

### 3.4 Microarray and RT-qPCR analyses in the liver

Western blot analysis of the liver revealed slightly attenuated responses to insulin in the phosphorylation level of Ser^473^ but not Thr^308^ of Akt in ArKO mice compared to those in WT mice. Next, we examined hepatic sensitivity to insulin at the transcriptional level using RT-qPCR.

**Fig. 4:**
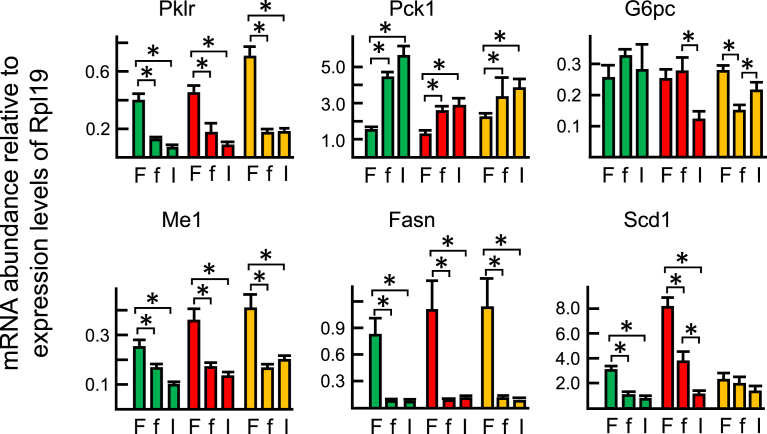
RT-qPCR analysis of genes related to gluconeogenesis and lipogenesis in the liver. Quantitative RT-PCR analysis of mRNA of genes related to gluconeogenesis (Pklr, Pck1, and G6pc) and lipogenesis (Me1, Fasn, and Scd1) were performed on cDNA derived from hepatic total RNA of WT (green bar), ArKO (red bar), and ArKO/E2 (orange bar) mice. Livers were collected from fed (F), fasted for 19 h (f), and at 60 min after insulin-injected mice (I). Expression data from 7 samples per experimental group are shown as the mean ± SD following normalization for ribosomal protein L19 mRNA expression. * indicates significant difference.

First, we examined whether the expression levels of genes relevant to glucose metabolism, such as pyruvate kinase (Pklr), phosphoenolpyruvate carboxykinase 1 (Pck1), and glucose-6-phosphatase catalytic subunit (G6pc), revealed the action of insulin in murine liver (Fig. 4). Transcripts of Pklr were significantly suppressed and those of Pck1 were induced by fasting in all experimental groups. No significant changes in the abundance of G6pc transcripts were observed in WT and ArKO mice by fasting, but a significant reduction was detected in ArKO/E2 mice. Insulin supplementation did not cause significant changes at least within the time frame we studied, except for the expression of G6pc in ArKO and ArKO/E2 mice, in which insulin significantly decreased and increased, respectively, G6pc mRNA abundance compared to the expression levels in fasted control mice. We further examined the expression levels of genes related to lipogenesis such as NADP (+)-dependent malic enzyme 1 (Me1), fatty acid synthase (Fasn), and stearoyl-coenzyme A desaturase 1 (Scd1). As observed for glucose metabolizing genes, fasting was associated with a significant decrease in mRNA abundance, except for Scd1 expression in ArKO/E2 mice where no significant changes were detected. Insulin supplementation did not significantly alter expression levels, except for Scd1 expression in ArKO mice. These results suggested that gluconeogenic and lipogenic genes were not relevant to examine differences in insulin responses between WT and ArKO mice.

**Fig. 5:**
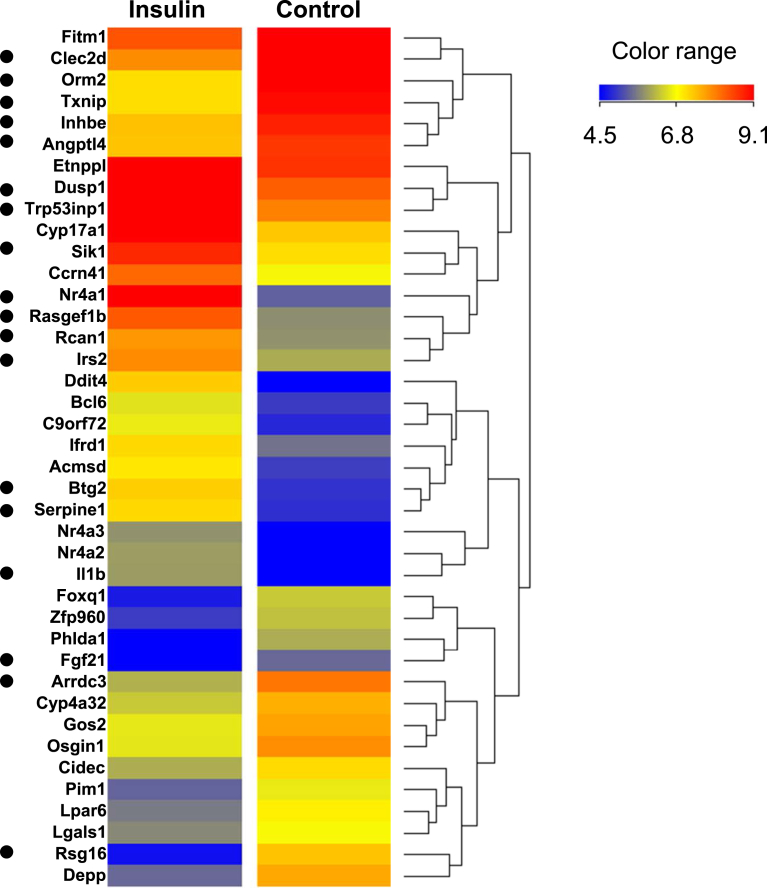
Heat map representing mRNA expression of the top 20 ranked insulin-target genes in the WT liver as determined by microarray analysis. A heat map was generated with Mev_4_8_1 after normalization of expression-values by Z score transformation. Genes selected for RT-qPCR analysis were marked with a dot. The color key indicates the correlation score: blue, lowest; red, highest.

Therefore, we conducted a microarray analysis to find indicators of insulin action under our experimental conditions. We identified 93 genes that had altered expression levels 1 h after insulin supplementation in fasted WT male mice; 62 genes were upregulated more than 2 fold and 31 genes downregulated less than 0.5 fold compared to those in PBS-administered WT mice. A heat map of the 20 top-ranked genes is shown in Fig. 5.

Based on the microarray analysis, we selected a subset of genes regulated by insulin in WT liver (10 upregulated genes: B cell translocation gene 2, anti-proliferative (Btg2), dual specificity phosphatase 1 (Dusp1), nuclear receptor subfamily 4, group A, member 1 (Nr4a1), interleukin 1 beta (Il1b), insulin receptor substrate 2 (Irs2), RasGEF domain family, member 1B (Rasgef1b), regulator of calcineurin 1 (Rcan1), serine (or cysteine) peptidase inhibitor, clade E, member 1 (Serpine1), salt inducible kinase 1 (Sik1), and transformation related protein 53 inducible nuclear protein 1 transcript variant 2 (Trp53inp1), and 9 downregulated genes: angiopoietin-like 4 (Angptl4), arrestin domain containing 3 (Arrdc3), C-type lectin domain family 2, member d (Clec2d), fibroblast growth factor 21 (Fgf21), fibrinogen-like protein 1 (Fgl1), inhibin beta-E (Inhbe), regulator of G-protein signaling 16 (Rgs16), orosomucoid 2 (Orm2), and thioredoxin interacting protein (Txnip)) and examined the expression levels in ArKO and ArKO/E2 mice by means of RT-qPCR.

**Fig. 6:**
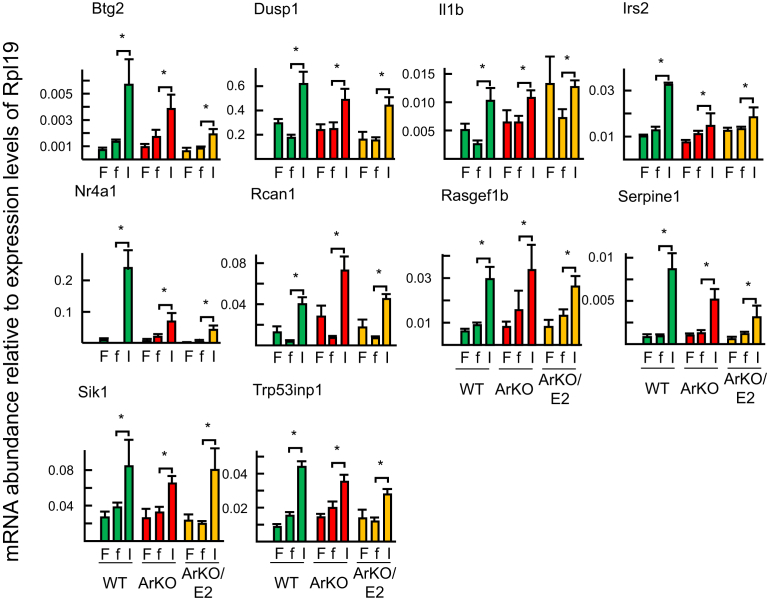
RT-qPCR analysis of genes upregulated by insulin. Livers were collected from fed (F), fasted (f), and at 60 min after insulin-injected fasted (I) WT (green bar), ArKO (red bar), and E2-supplemented ArKO (orange bar) mice. Expression data from 7 samples per experimental group are shown as the mean ± SD following normalization to ribosomal protein L19 mRNA expression. * indicates significant difference.

**Fig. 7:**
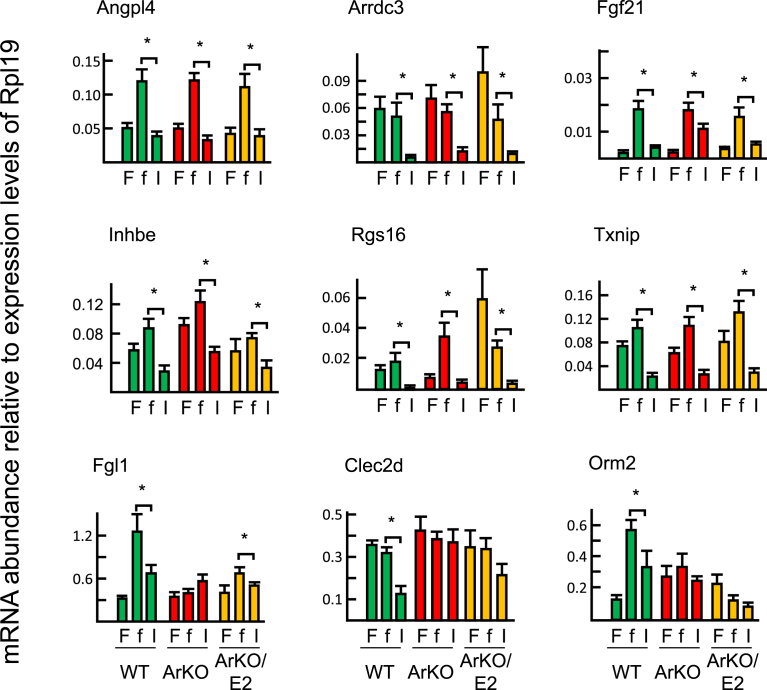
RT-qPCR analysis of genes downregulated by insulin. Livers were collected from fed (F), fasted (f), and at 60 min after insulin-injected fasted (I) WT (green bar), ArKO (red bar), and E2-supplemented ArKO (orange bar) mice. Expression data from 7 samples per experimental group are shown as the mean ± SD following normalization to ribosomal protein L19 mRNA expression. * indicates significant difference.

**Fig. 8:**
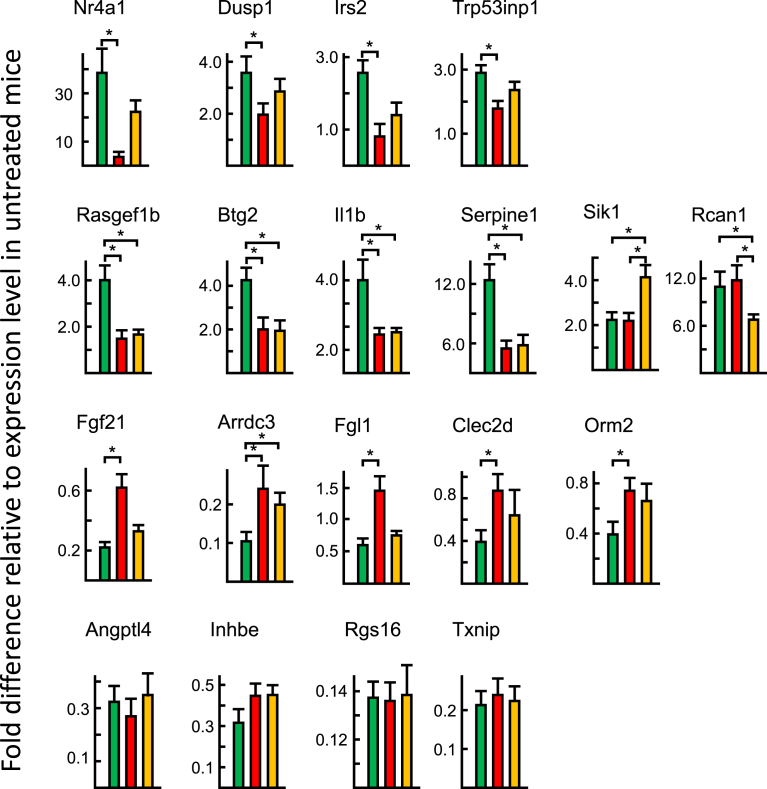
Analysis of the degrees of alterations by insulin on insulin-target genes by RT-qPCR analyses. Alterations in mRNA expression levels of each gene by insulin (Figs. 5 and 6) were calculated by the mean value of the expression level of fasted mice and compared among WT (green bar), ArKO (red bar), and ArKO/E2 (orange bar) mice. * indicates significant difference.

A significant induction of the 10 upregulated gene was detected in ArKO mice (Fig. 6). By contrast, 3 of 9 downregulated genes (Fgl1, Clec2d, and Orm2) were not suppressed by insulin in ArKO mice (Fig. 7). Although 16 of the 19 selected insulin-target genes were responsive to insulin in ArKO mice as observed in WT mice when analyzed by RT-qPCR, there were marked differences between WT and ArKO mice in the responses (Fig. 8). Namely 8 of 10 upregulated genes and 5 of 9 downregulated genes were significantly less altered in ArKO mice than in WT mice. E2 supplementation restored responses to WT levels in six genes (Nr4a1, Dusp1, Irs2, Trp53inp1, Fgf21, and Fgl1).

Taken together, data from RT-qPCR analysis revealed that ArKO mice were less responsive to insulin than WT mice, which may affect whole-body glucose homeostasis in ArKO mice.

## 4 Discussion

By means of two distinct approaches, we examined differences in insulin sensitivity between WT and ArKO mice: protein phosphorylation of Akt in the liver and muscle, and hepatic mRNA expression of insulin-target genes. Although suppression of endogenous glucose production by insulin is a well-established indicator to discriminate hepatic insulin resistance from extra-hepatic origins using a euglycemic-hyperinsulinemic clamp test, phosphorylation of Akt is also a sensitive and convenient indicator of insulin signaling under physiological conditions (Saltiel and Kahn, 2001; Whiteman et al., 2002).

Estrogen modulates insulin sensitivity and is involved in glucose homeostasis (Godsland, 2005). ArKO male mice progressively develop hyperglycemia and insulin resistance (Nemoto et al., 2000; Takeda et al., 2003). Because mice deleted the insulin receptor gene in a liver-specific manner have hyperglycemia and whole-body insulin resistance (Michael et al., 2000), we hypothesized that insulin-induced phosphorylation of Akt would be highly attenuated in the liver of ArKO mice compared to that in WT mice. However, we detected a significant but modest attenuation in the phosphorylation levels of Ser^473^ in Akt in ArKO liver compared to that of WT liver after insulin administration. By contrast, the phosphorylation levels of Ser^473^ were significantly higher in fasted ArKO mice compared to those in WT mice. These observations suggest that the functions of protein phosphatases (Newton and Trotman, 2014) or mammalian target of rapamycin complex 2 (mTORC2) responsible for phosphorylation of Ser^473^ in Akt (Sarbassov et al., 2005; Hagiwara et al., 2012) are dysregulated in estrogen-deficient conditions, which obscures insulin-dependent phosphorylation of Ser^473^ in Akt in the liver. In contrast to the liver, analysis of the gastrocnemius revealed a significant attenuation in the phosphorylation levels of both Ser^473^ and Thr^308^ in Akt in ArKO mice compared to those in WT mice in the fasted state. We also detected lower phosphorylation levels of Thr^308^ after insulin administration in ArKO mice in the muscle. Because skeletal muscle accounts for 70–90% of glucose disposal following a carbohydrate load (DeFronzo et al., 1981) through activation of Akt in an insulin-dependent manner (Jaldin-Fincati et al., 2017) and because estrogens increase insulin-stimulated glucose uptake into skeletal muscle *in vivo* (Gorres et al., 2011), low phosphorylation levels of Akt in muscle may reflect poor insulin sensitivity, thereby causing the development of hyperglycemia and insulin resistant conditions in ArKO mice. Nevertheless, the relationship between Akt phosphorylation levels and insulin activity is tenuous because Akt is reported to be dispensable for insulin-mediated metabolic regulation (Okamoto et al., 2005; Lu et al., 2012). Furthermore, other studies reported that differences in phosphorylation levels of Akt between WT and ArKO mice may occur only in female mice (Van Sinderen et al., 2014, Van Sinderen et al., 2015), though experimental conditions between those studies and ours, such as the dosage of insulin (0.15 U/g body weight (Van Sinderen et al., 2014) and 0.75 mU/g body weight in this study) and procedures for insulin administration (via inferior vena cava versus ip), were slightly different.

E2 supplementation in ArKO mice reverses hyperinsulinemia and hyperglycemia to WT levels (Fig. 1) and improves the impairment of glucose disposal activity (Takeda et al., 2003; Van Sinderen et al., 2014). We observed improvement in the phosphorylation levels of Ser^473^ in Akt in the liver of ArKO/E2 mice 10 min after insulin stimulation compared to that of ArKO mice, which suggests that lowered insulin sensitivity from estrogen deficiency was corrected by E2 supplementation. The marked positive effect of E2 on insulin-stimulated phosphorylation of Akt was also observed at Thr^308^ in the liver. E2 supplementation resulted in enhancement of phosphorylation levels of Akt in the muscle after insulin administration: nevertheless the difference was not significant. Thus, the effect of E2 on Akt phosphorylation seems to correlate with the improvement of whole-body glucose metabolism in ArKO mice treated with estrogen. However, treatment of rat soleus muscles with E2 in vitro increases the phosphorylation level of Ser^473^ in Akt (Rogers et al., 2009), indicating that E2 enhances phosphorylation of Akt without insulin treatment. This effect of E2 on Akt phosphorylation might explain high phosphorylation levels of Akt at Ser^473^ observed in the liver and muscle of fasted ArKO/E2 mice. We also detected an increment in the phosphorylation levels at Thr^308^ of Akt in the muscle of fasted ArKO/E2 mice compared to those of ArKO mice, but not in the liver. The Thr^308^ of Akt is proposed to be phosphorylated through the phosphoinositide 3-kinase (PI3K) dependent pathway (Manning and Toker, 2017). Thus E2 might generate only a modest effect on the PI3K-Akt pathway, partly through the G-protein coupled estrogen receptor (Revankar et al., 2005; Kim and Bender, 2009; Petrie et al., 2013), in the ArKO liver.

Insulin stimulates many signaling pathways, including the Ras/MEK/ERK pathway, which lead to initiation of a transcriptional program (Boulton et al., 1991). Under our experimental conditions, we were unable to detect insulin's ability to suppress and enhance gene expression related to hepatic glucose production and fatty acid synthesis, respectively, in WT mice. To examine hepatic sensitivity to insulin transcriptionally, we employed microarray analysis. We identified insulin-target genes and selected 19 genes for analysis. Of the 19 genes, six genes (Rasgef1, Serpine1, Trp53inp1, Fgl1, Clec2d, and Orm2) are novel insulin-regulatable genes in the liver. The responses to insulin of eight genes, Dusp1 (Lawan et al., 2015), Il1b (Lang and Dobrescu, 1989), Rcan1 (Wang et al., 2012), Sik1 (Berdeaux, 2011), Angpl4 (Oike et al., 2005), Arrdc3 (Patwari et al., 2011), Rgs16 (Huang et al., 2006), and Txnip (Chutkow et al., 2008), were consistent with previous studies. However, the expression of Nr4a1 and Btg2 genes was previously reported to be regulated by glucagon (Pei et al., 2006; Kim et al., 2014), but we found a significant induction by insulin, which suggests that expression of these genes was regulated by insulin as well as glucagon. FGF21 is a metabolic hormone with pleiotropic effects on glucose and lipid homeostasis (Erickson and Moreau, 2017). We found that expression of the Fgf21 gene was increased by fasting and significantly decreased by insulin, which is consistent with a previous report (Uebanso et al., 2009) but differs from a report that found that insulin increased Fgf21 mRNA abundance in rat hepatocyte cultures (Cyphert et al., 2014). Furthermore, the mRNA abundance of Irs2 and Inhbe was increased and decreased, respectively, after insulin administration under our experimental conditions, but opposite results have been reported for each gene (Hirashima et al., 2003; Hashimoto et al., 2009). Apparently further studies are needed to clarify the reasons why the contrasting responses to insulin were observed and how this disparity relates to glucose metabolism in vivo.

The mRNA levels of 10 selected genes induced by insulin in WT liver were also increased in ArKO liver, but 3 of 9 genes (Clec2d, inhbe, and Orm2) suppressed by insulin in WT liver were not suppressed in ArKO liver. These findings suggest that the insulin signaling pathway that suppresses target genes may be less functional in ArKO mice than in WT mice. These results resemble a characteristic of insulin resistance known as the “insulin signaling paradox” (Brown and Goldstein, 2008; Williams et al., 2013), which is characterized by enhanced lipid synthesis and defective suppression of glucose production in response to insulin in the liver. When insulin-induced gene alterations were assessed, less marked alterations were detected in ArKO livers compared to those in WT livers. Thus, ArKO liver seems to be insulin resistant from a transcriptional point of view.

E2 supplementation in ArKO mice did not reverse the phosphorylation levels of Thr^308^ of Akt in the muscle and the expression levels of all insulin-target genes to the WT levels, nevertheless blood glucose and insulin levels in fasted state are completely normalized under the current E2 supplementation conditions. This might reflect that conditions for E2 supplementation after maturation are inappropriate. It is also possible that E2 levels might be inappropriate to regulate subsets of E2-dependent physiology in E2-supplemented ArKO mice. Determination of E2 levels in the E2-target tissues such as liver and muscle in addition to serum of WT, ArKO and ArKO/E2 mice is essential to clarify whether the observed effects of E2 in the present study are due to pharmacological levels of E2 or not. Nevertheless, determination of serum E2 concentrations in male mice seems to be a challenging area, partly because concentrations of estrogenic steroids are below the limit of detection (<0.3 pg/ml for E2 and <0.5 pg/ml for estrone) by gas chromatography-tandem mass spectrometry (Nilsson et al., 2015) or because of inability of the quantification due to unknown reasons by liquid chromatography-tandem mass spectrometry (McNamara et al., 2010).

In conclusion, we found lower sensitivity of gastrocnemius in ArKO mice to insulin in terms of phosphorylation levels of Akt than that of WT mice, but the responses in the liver were relatively comparable between ArKO and WT mice. However, from a transcriptional point of view, the liver of ArKO mice was less sensitive to insulin than that of WT mice, and the sensitivity of ArKO mice was partially restored by E2 supplementation, although it remains to be determined whether or not the observed effects of E2 in ArKO male mice are attributable to the pharmacological influence of E2. Taken together, our present study demonstrated that attenuated responses to insulin might cumulatively affect glucose metabolism and lead to hyperglycemia in ArKO mice.

## Declarations

### Author contribution statement

Katsumi Toda: Conceived and designed the experiments; Performed the experiments; Analyzed and interpreted the data; Contributed reagents, materials, analysis tools or data; Wrote the paper.

Akiko Toda: Performed the experiments.

Masafumi Ono: Performed the experiments; Analyzed and interpreted the data.

Toshiji Saibara: Conceived and designed the experiments; Performed the experiments; Analyzed and interpreted the data; Wrote the paper.

### Funding statement

This work was supported in part by a grant in aid for Scientific Research from the Ministry of Education, Culture, Sports, Science and Technology, Japan (to T. Saibara, no. 15K09010).

### Competing interest statement

The authors declare no conflict of interest.

### Additional information

Data associated with this study has been deposited online at Gene Expression Omnibus (www.ncbi.nlm.nih.gov/geo) under the accession number GSE111407.
