# Supplementary material for: Lack of 17β-estradiol reduces sensitivity to insulin in the liver and muscle of male mice
Source: Heliyon. 2018 Sep 11;4(9):e00772. doi: 10.1016/j.heliyon.2018.e00772 (PMC6134327; doi:10.1016/j.heliyon.2018.e00772)
Supplement: Supplementary Figure 1 [file mmc1.pdf]

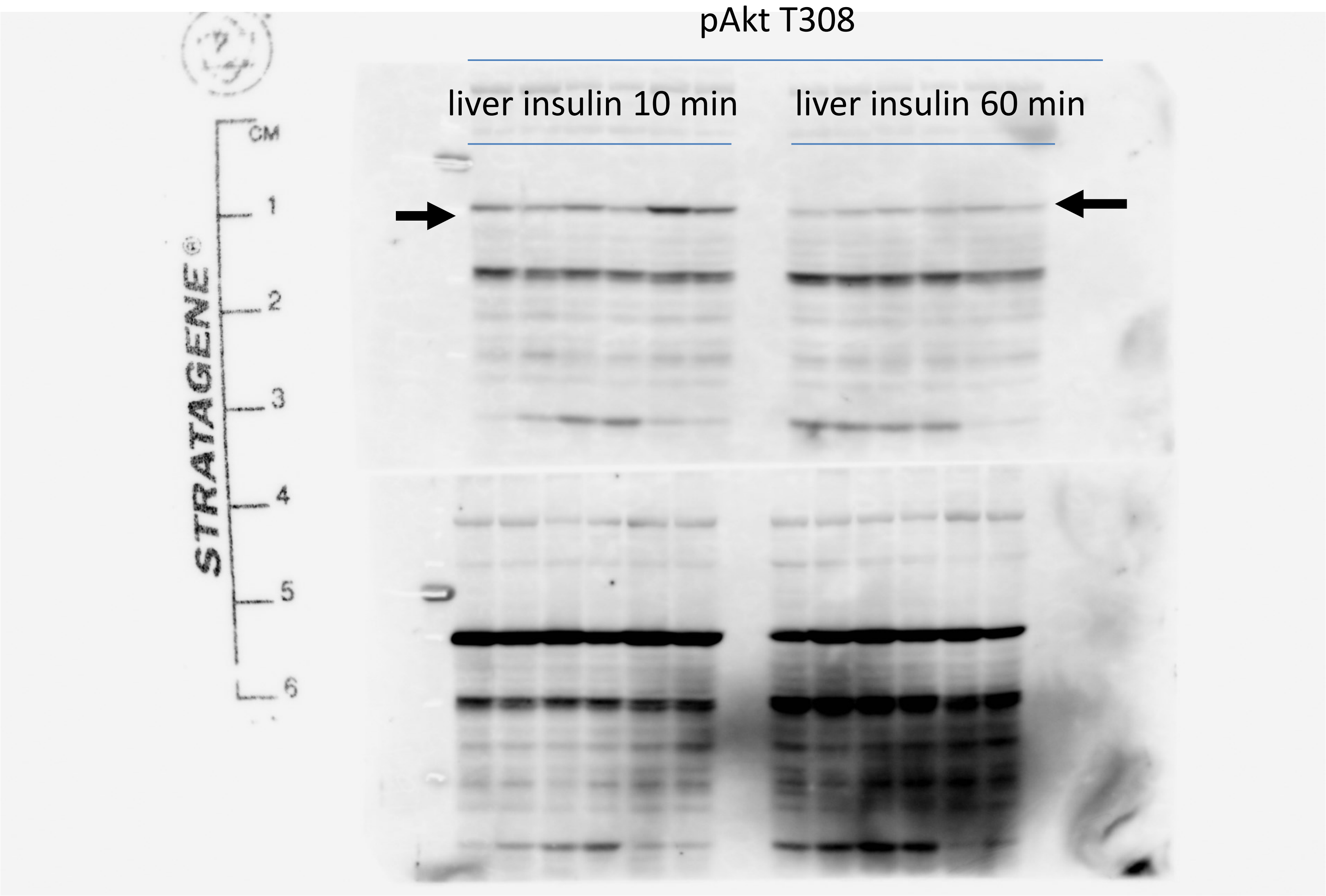
pAkt T308
liver insulin 10 min
liver insulin 60 min
STRATAGENE
CM
1
2
3
4
5
6

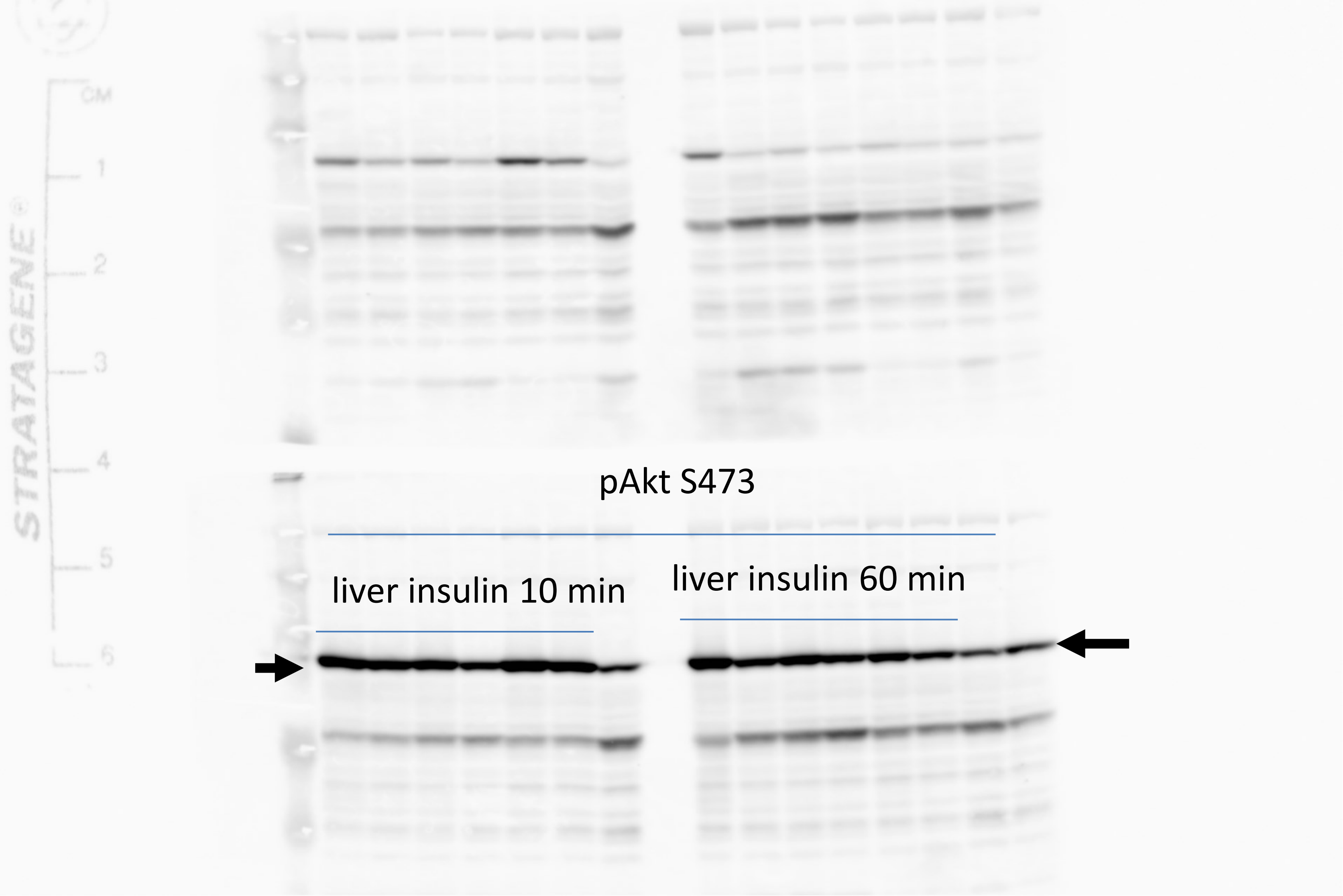

STRATAGENE
CM
1
2
3
4
5
6
pAkt S473
liver insulin 10 min
liver insulin 60 min

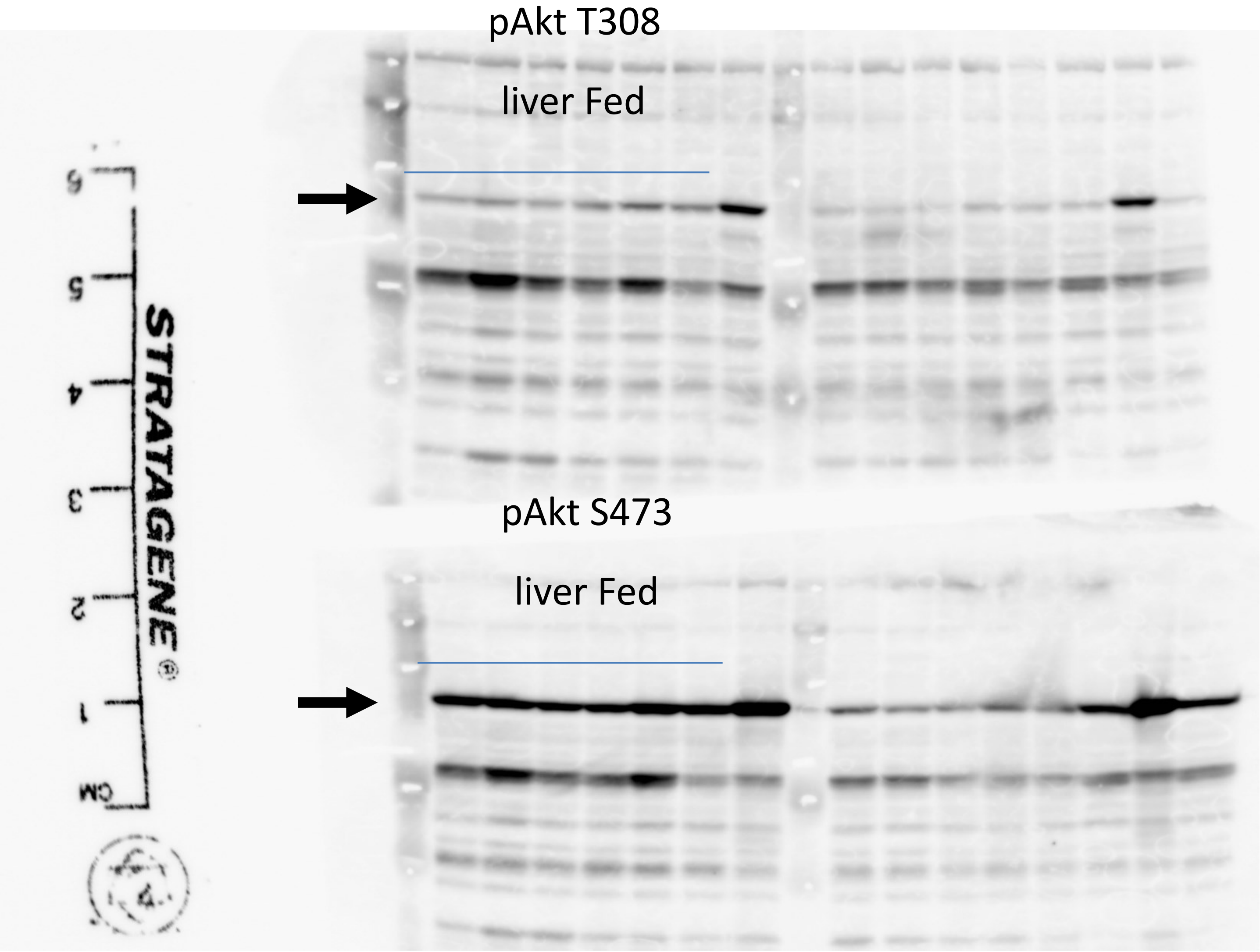
pAkt T308
liver Fed
pAkt S473
liver Fed
STRATAGENE

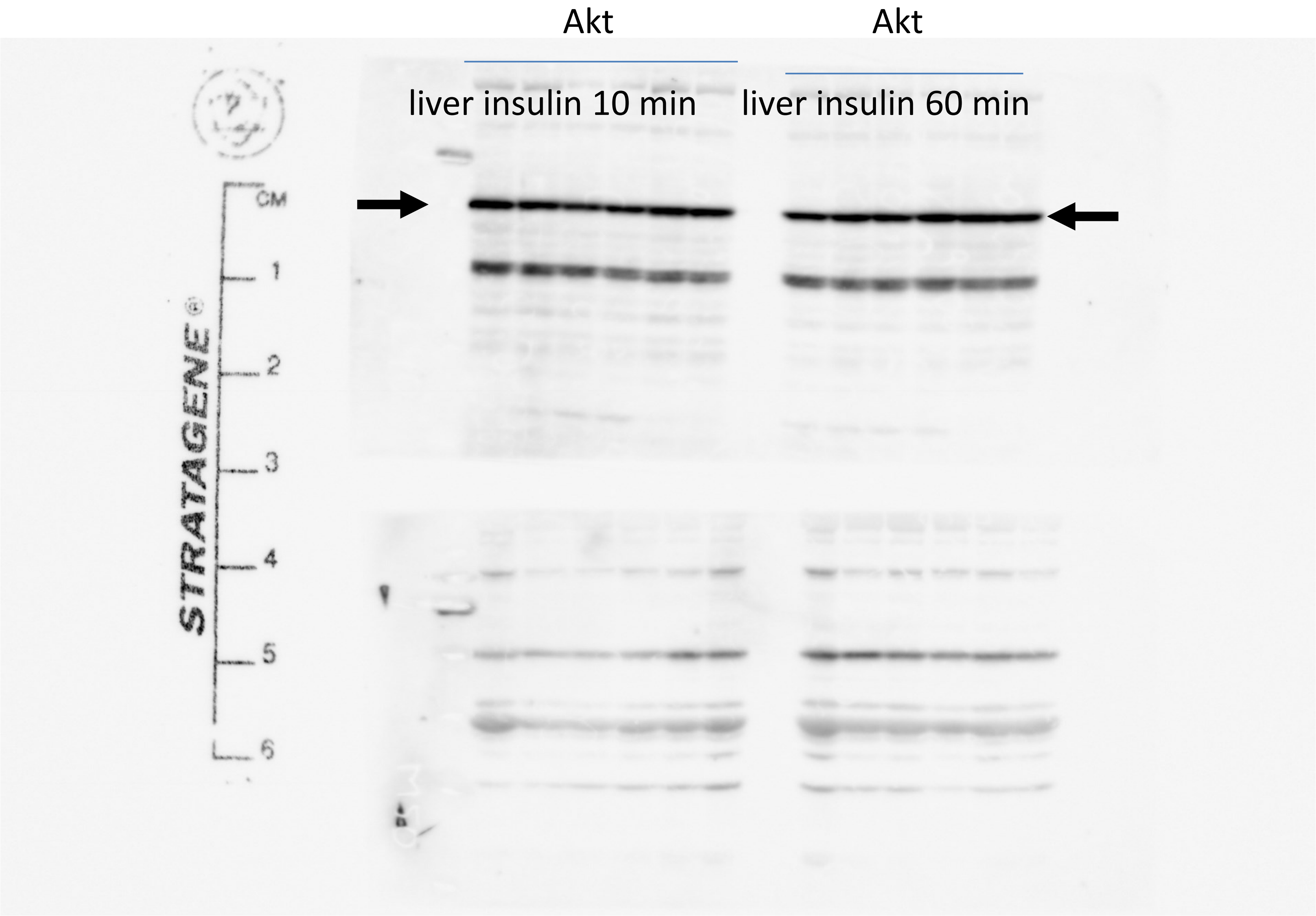
Akt
liver insulin 10 min
Akt
liver insulin 60 min
STRATAGENE®
CM
1
2
3
4
5
6

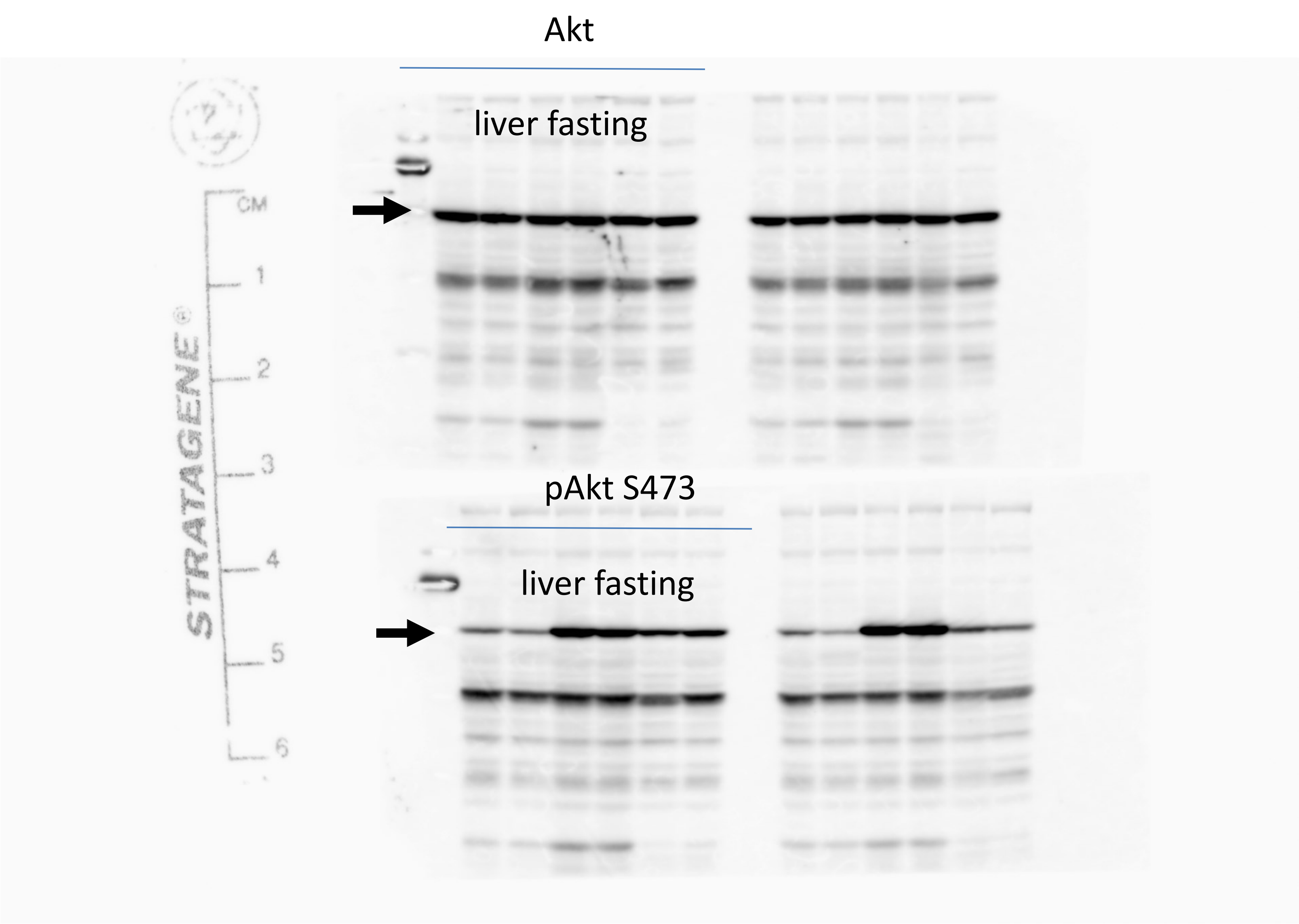

Akt
liver fasting
pAkt S473
liver fasting
STRATAGENE
CM
1
2
3
4
5
6

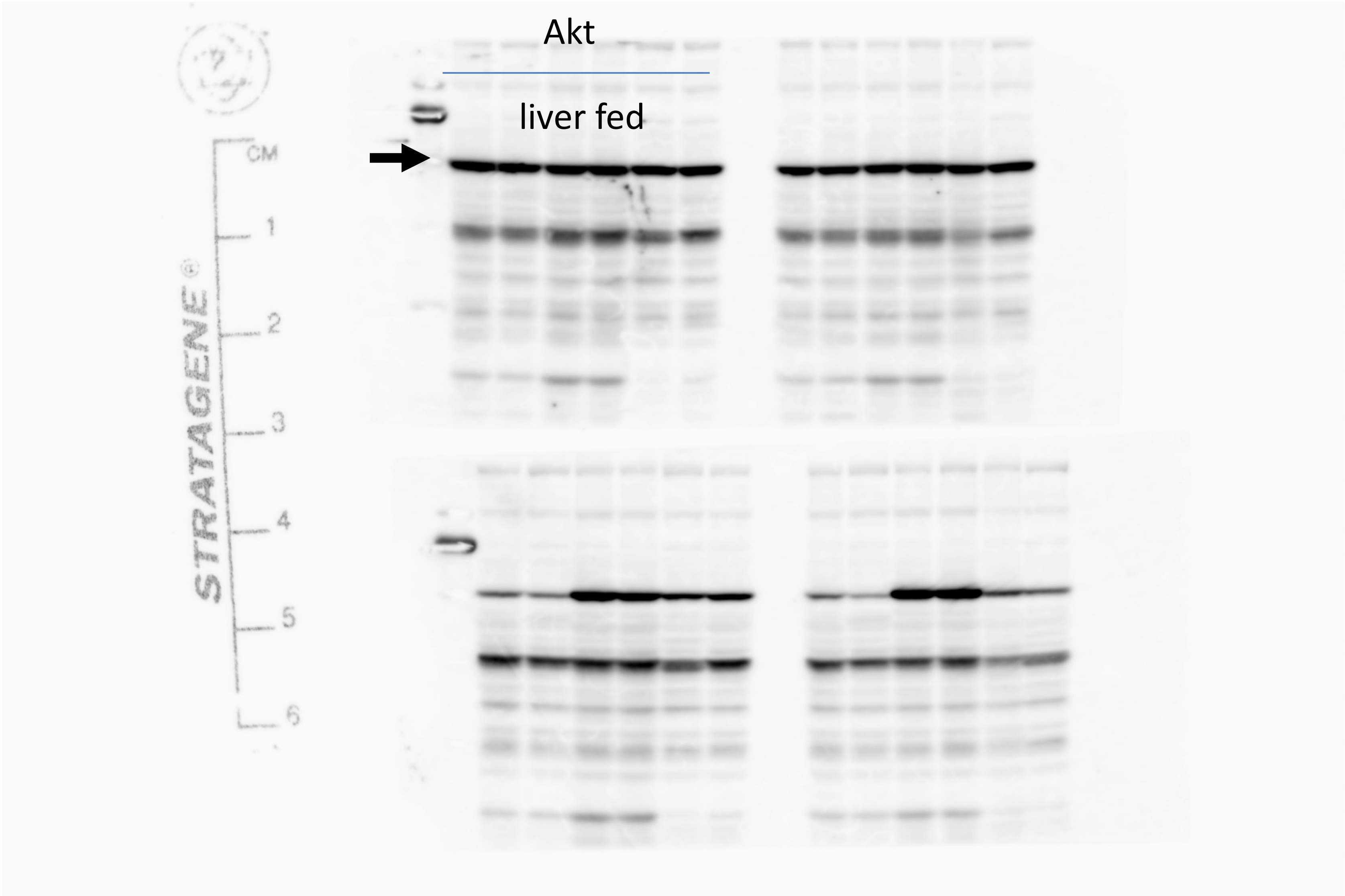
Akt
liver fed
STRATAGENE
CM
1
2
3
4
5
6

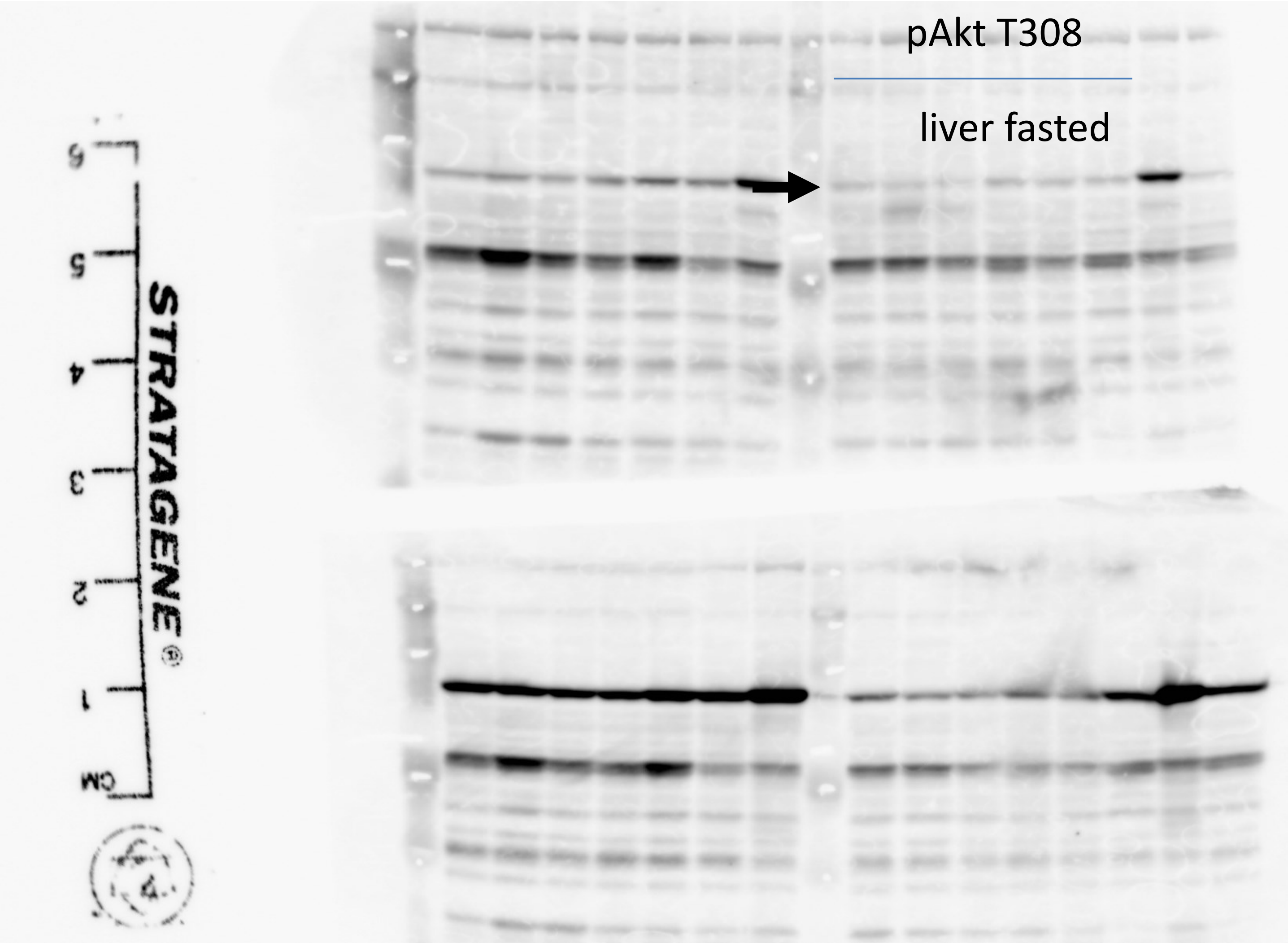

pAkt T308
liver fasted
STRATAGENE
