# Supplementary material for: Lack of 17β-estradiol reduces sensitivity to insulin in the liver and muscle of male mice
Source: Heliyon. 2018 Sep 11;4(9):e00772. doi: 10.1016/j.heliyon.2018.e00772 (PMC6134327; doi:10.1016/j.heliyon.2018.e00772)
Supplement: Supplementary Figure 2 [file mmc2.pdf]

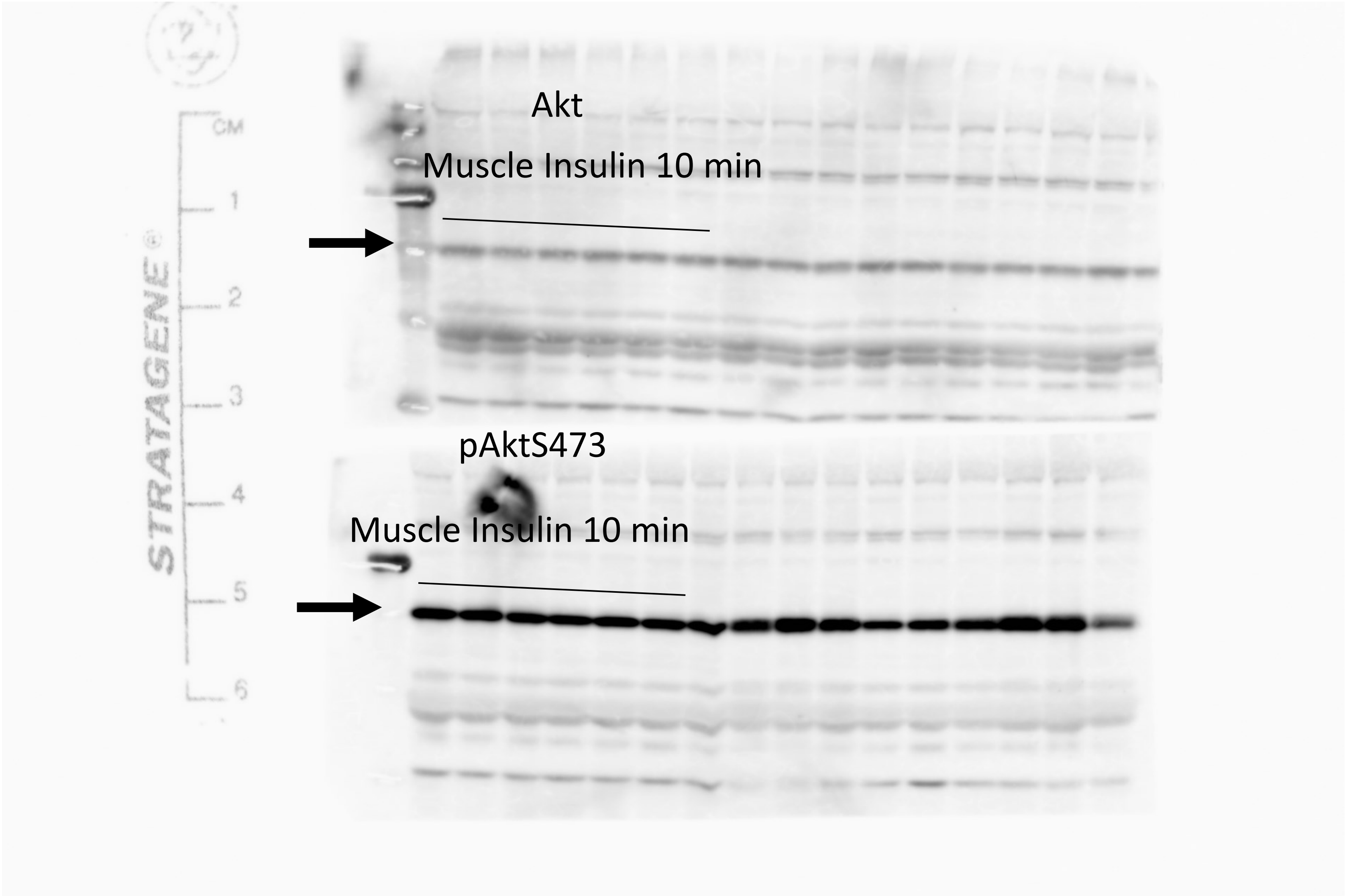

Akt
Muscle Insulin 10 min
pAktS473
Muscle Insulin 10 min
STRATAGENE®
CM
1
2
3
4
5
6

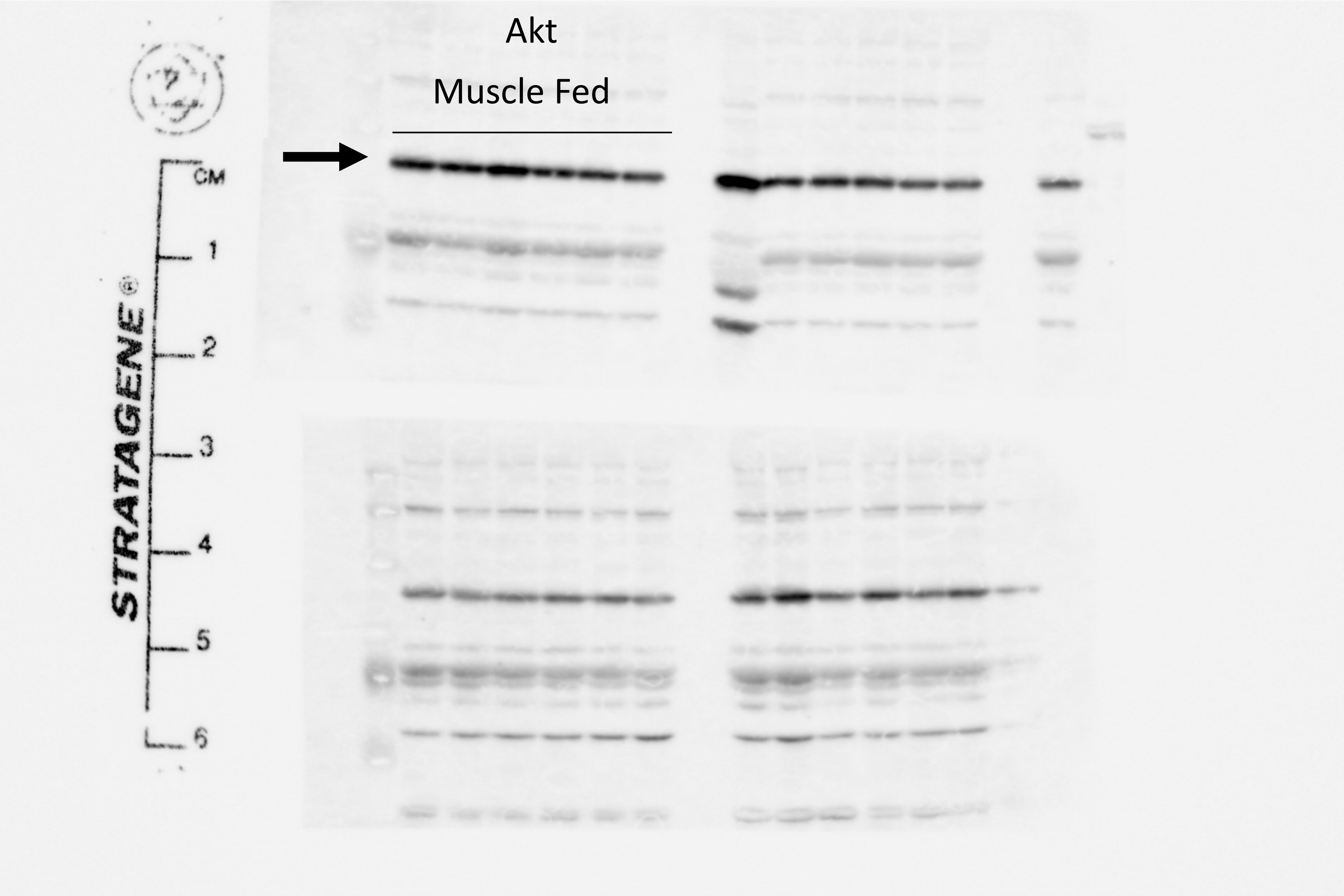
Akt
Muscle Fed
STRATAGENE®
CM
1
2
3
4
5
6

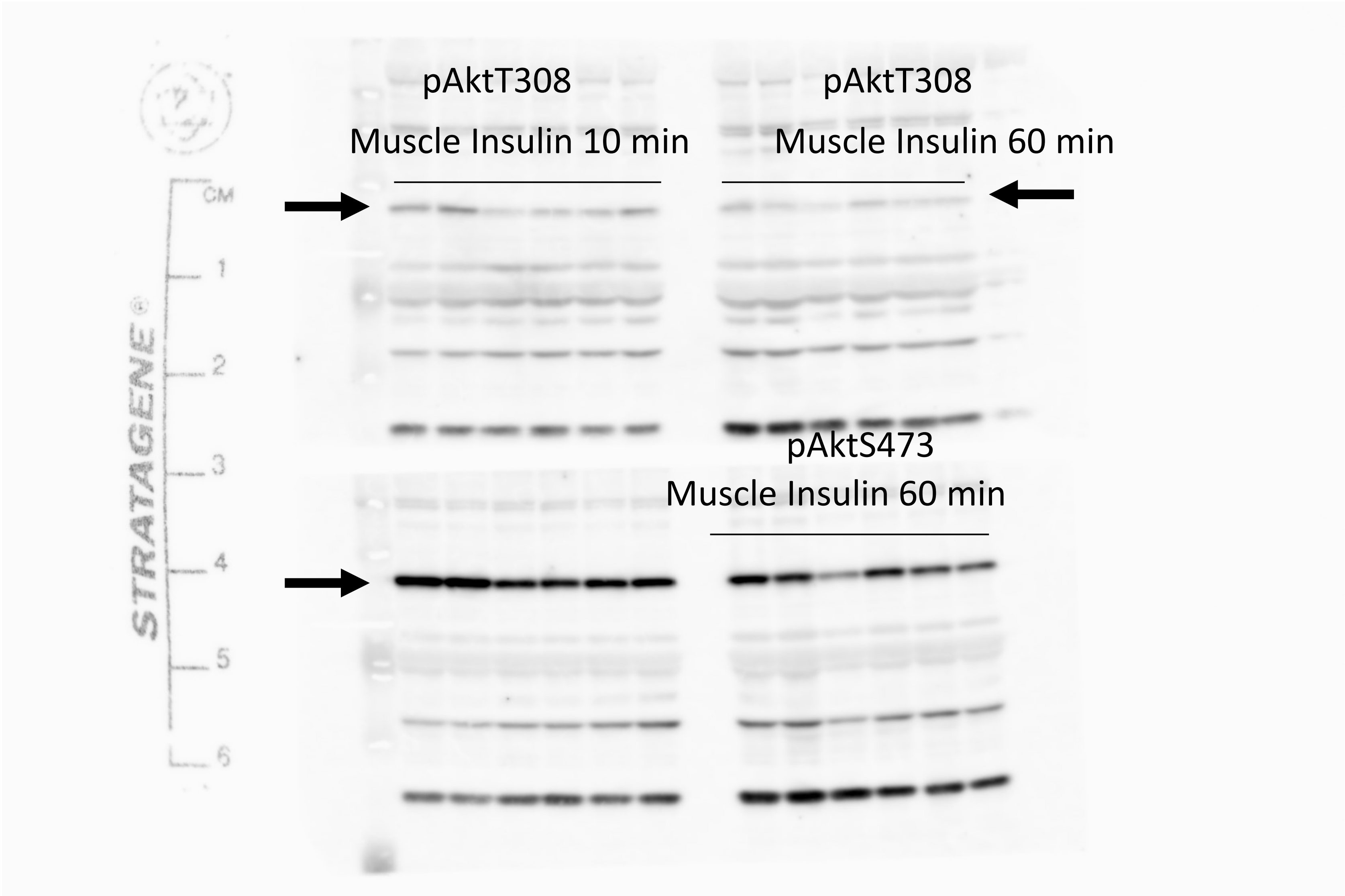
pAktT308
Muscle Insulin 10 min
pAktT308
Muscle Insulin 60 min
pAktS473
Muscle Insulin 60 min
STRATAGENE
CM
1
2
3
4
5
6

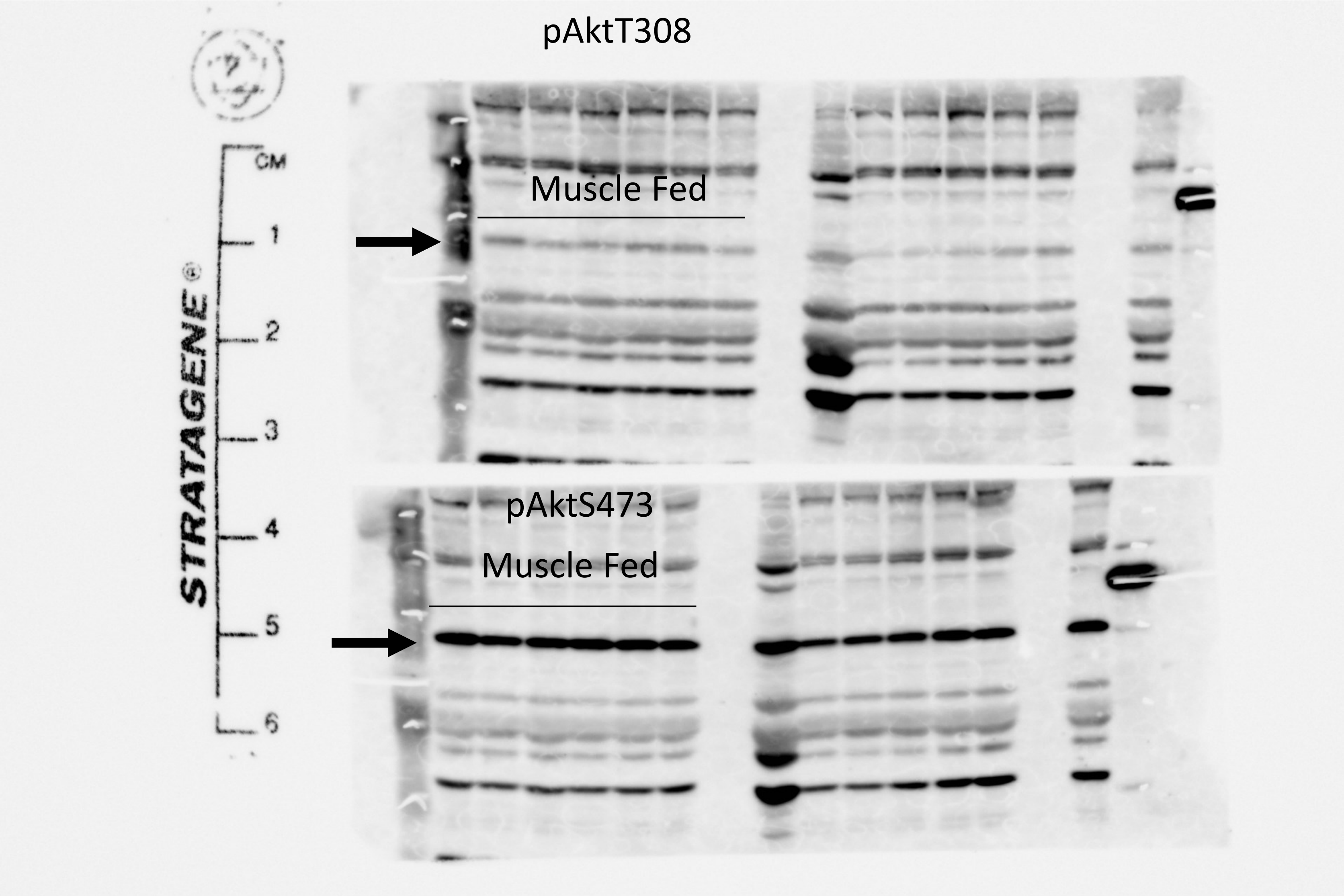
pAktT308
Muscle Fed
pAktS473
Muscle Fed
STRATAGENE®
CM
1
2
3
4
5
6

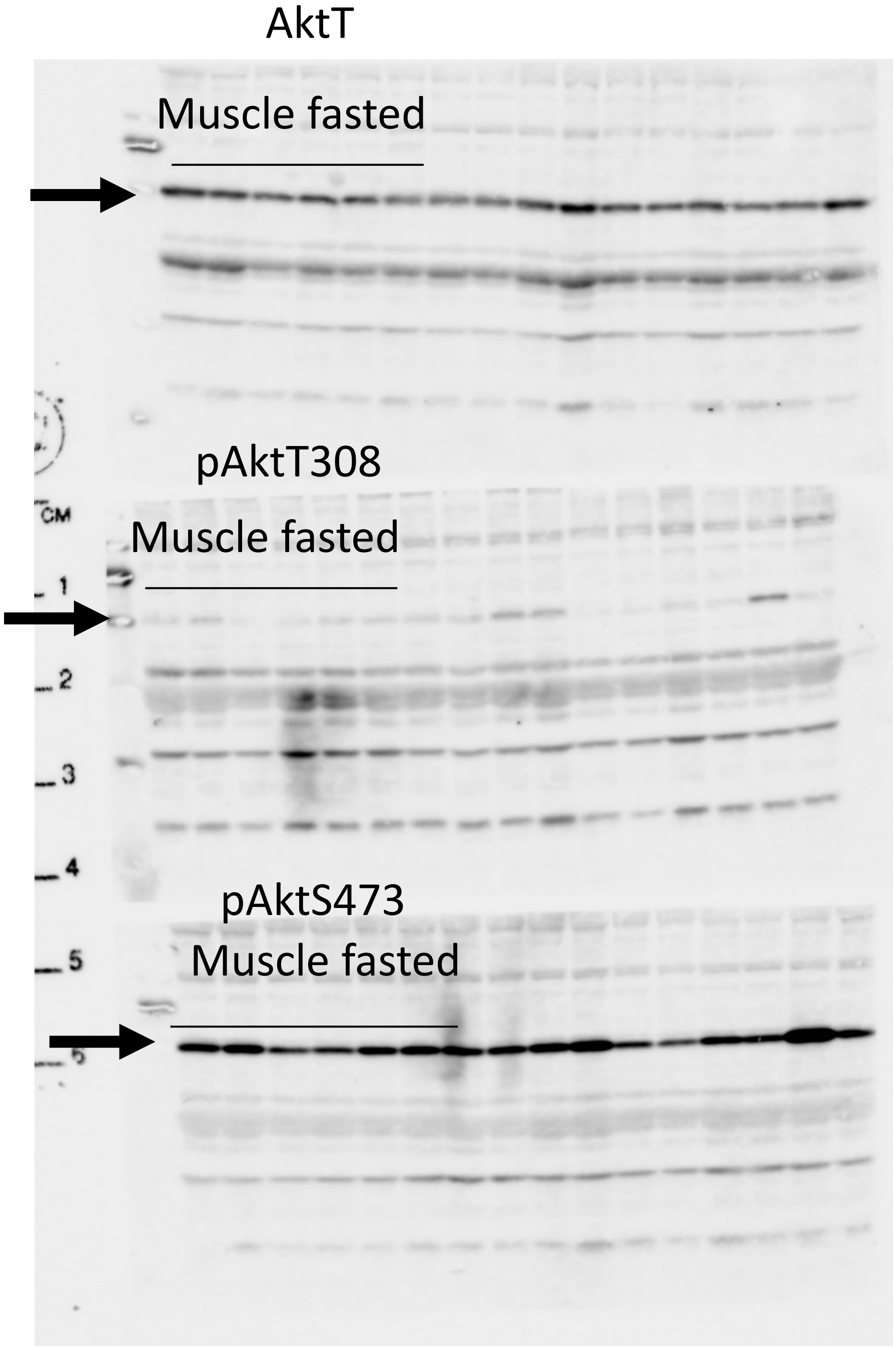
AktT
Muscle fasted
pAktT308
Muscle fasted
pAktS473
Muscle fasted

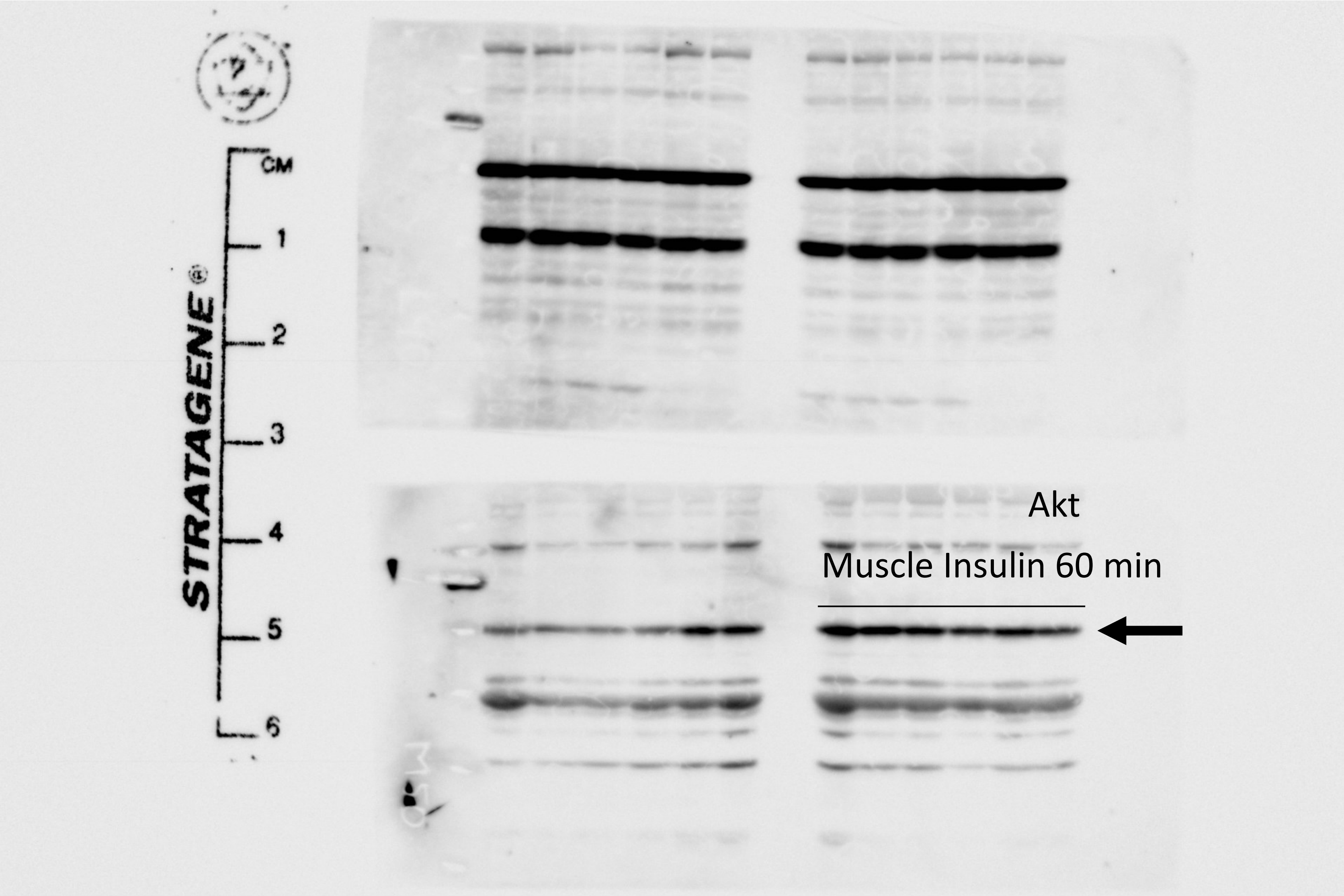

STRATAGENE®
CM
1
2
3
4
5
6
Akt
Muscle Insulin 60 min
